# A Comparison of Strategies to Improve Population Diets: Government Policy versus Education and Advice

**DOI:** 10.1155/2020/5932516

**Published:** 2020-06-04

**Authors:** Norman J. Temple

**Affiliations:** Centre for Science, Athabasca University, Athabasca, Alberta T9S 3A3, Canada

## Abstract

Different strategies have been utilized in order to improve the healthiness of the population diet. Many interventions employ education, advice, and encouragement (EAE). Those interventions have been carried out in diverse settings and may achieve modest success; the estimated risk of cardiovascular disease is lowered by about 5–15%. An alternative strategy is action policies carried out by the governments. The removal of trans-fatty acids from food is a model for a successful action policy. Other action policies include requiring a substantial reduction in the amount of salt added to processed foods and ordering schools to cease supplying unhealthy food to students. Taxes and subsidies can be used to increase the price of unhealthy foods, such as sugar-rich foods, and reduce the price of healthy foods, such as fruit and vegetables. It is very probable that action policies are more effective than those based on EAE. They are also much more cost-effective.

## 1. Introduction

Strong evidence has emerged since the 1970s that many chronic diseases are closely related to the Western lifestyle and are therefore potentially preventable [1]. This family of diseases is now commonly known as chronic diseases of lifestyle (CDL). These hugely important findings triggered an enormous effort to discover effective interventions that would persuade the population to adopt a healthier lifestyle so as to enhance health and prevent disease. These diverse interventions have led to the emergence of health promotion as a distinct field.

This paper summarizes the key lessons learned from 40 years of health promotion interventions with particular reference to nutrition. Health promotion interventions are divided into two broad types:

  Education, advice, and encouragement, with the goal of persuading people to adopt a healthier diet;
  Action policies carried out by governments, such as manipulating food prices or reformulating food.

The main focus of this paper is a comparison of the effectiveness of the two types of health promotion.

## 2. Health Promotion Interventions: Those Based on Education, Advice, and Encouragement

Many health promotion interventions have been carried out using diverse approaches to education, advice, and encouragement (EAE). One such type is a community intervention. Three major projects were carried out in the USA during the 1980s. These were the Stanford Five-City Project in California [2], the Minnesota Heart Health Program [3], and the Pawtucket Heart Health Program in Pawtucket, Rhode Island [4]. A wide variety of methods were used, such as information in the mass media, schools, and supermarkets, in order to deliver EAE to the target population. The goals were to lower elevated levels of blood cholesterol, blood pressure, and body weight, to cut smoking rates, and to persuade people to engage in exercise. These ambitious projects lasted between five and eight years. An analysis of the combined results of the three projects revealed very little improvement in blood cholesterol, blood pressure, body weight, and smoking [5]. Reflecting these disappointing findings, there was no change in the estimated risk of coronary heart disease (CHD).

Clearly, the above three community interventions were dismal failures. Many other community interventions have been carried out in the years since those three projects. Fortunately, some modest success has been achieved. A systematic review of interventions that were designed to prevent cardiovascular disease (CVD) came to the following conclusions [6]. Overall, systolic blood pressure was reduced by 2.9 mm Hg, total cholesterol level by 0.01 mmol/L, and smoking prevalence by 1.7%. The authors estimated that these changes will reduce the 10-year risk of CVD by 9.1% (reduction relative to baseline).

Worksites are another common target for health promotion interventions. A variety of strategies have been employed in order to encourage workers to eat a healthier diet, such as point-of-purchase food labelling and increasing the availability of fresh fruits and vegetables. Overall, the findings suggest that these interventions can often achieve a modest improvement in the diet [7, 8]. Unfortunately, firm conclusions cannot be made as many studies had weaknesses in the methodology.

Health promotion interventions have also been carried out in medical settings such as a physician's office. A review of 32 intervention studies reported that they achieved a modest but statistically significant impact on physical activity, intake of dietary fat, body weight, blood pressure, and blood cholesterol [9]. The authors concluded that “whereas small by conventional statistical definitions, these findings are likely to be meaningful when considered from a public health perspective.”

It is well established that people on a low income typically have the least healthy diets and the highest rates of CDL. This section of the population should therefore be a priority target for intervention. An assessment of the impact of behavior interventions on the diets of low-income people concluded that these interventions often achieve a small improvement in diet that is equivalent, on average, to eating just under half a portion of fruit and vegetables more per day [10].

Food labels should, ideally, provide shoppers with useful information on the nutritional value of the food while also being easy to understand. The most common label on food packages is the back-of-package label. This lists the major ingredients in the food and its content of calories and some key nutrients. The design of these labels leaves much to be desired as a great many shoppers find them hard to understand [11].

In an effort to rectify this problem, many countries have adopted front-of-package (FOP) labels. These are intended to be easy for shoppers to understand and clearly explain whether or not the food is healthy. There are three distinct designs: (1) some include summary information on the food content of three or four substances (e.g., traffic lights and guideline daily amounts); (2) some summarize the overall health value of the food using a symbol (such as stars or a tick); (3) and some provide a symbol to warn shoppers that the food has an excessive content of substances that are unhealthy when consumed in excess, especially sugar and salt. Many studies indicate that FOP labels can enhance the ability of shoppers to distinguish between more healthy and less healthy foods [12]. Studies have also reported that exposure to FOP labels often results in shoppers displaying an increased intent to buy healthier foods. Warning labels are the design that is most likely to persuade shoppers that they should reject unhealthy foods [12].

Unfortunately, these studies may have a serious flaw as they were carried out using a simulated shopping situation, mostly on a computer. We cannot assume that the findings from such studies reflect the behavior of shoppers in real-world supermarkets. Fortunately, a few studies have been carried out in real-world supermarkets with shoppers studied over several weeks or months. The findings indicate that FOP labels (or similar labels attached to shelves adjacent to the foods) probably induce a small improvement in the healthiness of food purchases, but the magnitude of the change is probably no more than 2% [12]. A reasonable conclusion from these studies is that FOP labels are of some limited value but are unlikely to lead to a significant improvement in the national diet.

Food guides are another tool in the general area of EAE. Dozens of governments have published food guides in order to advice their citizens on how to construct a healthy diet. There are many different designs in use [13]. How effective are they at leading to improvements in the diets eaten by populations? There is little reliable information on this, but we can make an inference from the above-mentioned findings on FOP food labels. Those labels lead to only a very small improvement in the diet consumed by the general population. Bearing in mind that FOP labels are present on a great many different food packages and are designed so that they are easy to see, we can reasonably conclude that food guides, which are very easy for people to ignore, will have even less impact on the national diet.

People are exposed to large numbers of advertisements on TV designed to persuade viewers to buy unhealthy (junk) foods. Such advertising is often successful in inducing children to consume the advertised foods [14, 15] and is strongly associated with the risk of obesity in children and adolescents [16]. It follows, therefore, that it makes perfect sense to implement a policy banning adverts that encourage young people to eat unhealthy food. This can be seen as the mirror image of EAE. Evidence suggests that such a ban is an effective means to decrease consumption of unhealthy food by young people. It has been estimated that such a ban on TV advertising in the USA would lead to an approximate 19% decrease in the numbers of children and adolescents eating two or more fast food meals per week [17]. This is predicted to reduce obesity rates by nearly one percentage point (though others investigators have reported higher estimates) [17]. This level of change is certainly helpful but is still quite modest.

## 3. How Successful Is Education, Advice, and Encouragement?

What lessons can be drawn from health promotion interventions that have centered around EAE? A wide variety of such interventions have been carried out over the last 30 years. Findings have been mixed: some studies have had essentially no impact whereas others have achieved a moderate degree of success. Changes in target measures have typically been no more than a few percentage points. Looking at these interventions as a whole, risk of CVD is likely to be reduced by about 5% to 15%. This is obviously a valuable contribution to public health, but this still leaves most of the population unaffected. Thus, exhortations to the individual, whether via the media, in the community, at the worksite, or in the physician's office, cannot prevent the large majority of cases of CDL.

FOP labels are comparable to the above in their degree of success. There is clear evidence that they enable shoppers to distinguish between more healthy and less healthy foods. However, studies carried out in real-world supermarkets suggest that FOP labels have very little positive impact on the healthiness of people's diets.

One type of EAE that does appear to lead to a significant shift towards healthier diets is the introduction of regulations that curtail the advertising of unhealthy food to young people.

This brief summary of health promotion strategies strongly suggests that they achieve only a small degree of success at improving the population diet. Another line of evidence pointing to the limited impact of EAE on the diet of the general population is to look at the extent to which the population has adopted a healthy diet. Over the last few decades, an enormous amount of general dietary advice has been delivered to the populations of all Western countries. The overall picture is that improvements in the diet have been disappointing. This is demonstrated by examining recent surveys of the diet eaten by the population of the USA [18]. There have been some encouraging trends, such as a significant decrease in intake of added sugar during the period 1999 to 2012. However, such improvements are overshadowed by poor progress elsewhere:

  Only 21.5% of the population eats one or more servings each day of whole fruit
  Americans eat, on average, 5.3 servings per day of refined grains but only one serving of whole grains

Arguably, the failure of decades of general dietary advice to persuade the population to switch to a healthy diet is best exemplified by the following findings. Surveys carried out in the USA and UK reveal that almost 60% of calorie intake comes from ultraprocessed foods, i.e., mixtures of such ingredients as sugar, refined flour, and added fats, plus liberal amounts of salt [19, 20]. The assertion made here that health promotion strategies have achieved only limited success at improving the population diet must be viewed with caution. Many factors may affect population diets including the rising popularity of dining out, economic changes (e.g., lingering effects of the great recession of 2008–2009), and rising inequality.

The evidence discussed here, taken as a whole, strongly suggests that EAE has only a modest impact on population diet and risk of CDL. Many different types of studies have been referred to (such as health promotion interventions and studies of whole populations) as well as a variety of outcomes (such as change in diet and change in estimated risk of CVD). Each type of study and each measurement of an outcome have inherent sources of error. For that reason, the limitations of the supporting evidence must be stressed. Caution must, therefore, be exercised before drawing firm conclusions.

## 4. Health Promotion Interventions: Those Based on Action Policies

There is a compelling case that EAE has a modest, but limited value: it is unlikely to lead to a major improvement in the population diet. Attention is now turned to an alternative strategy in order to improve the nutrition quality of the diets consumed by the population, namely, the interventions carried out by governments using proactive approaches such as interventions designed to change food prices and implementing policies that force food manufacturers to make food healthier. This strategy is referred to here as action policies.

Action policies by governments in the area of public health have a long history. Many such policies were implemented in the nineteenth century and achieved great success. Deadly infectious diseases, such as cholera and typhoid, were brought under control by such measures as providing the population with safe drinking water as well as sewage disposal. This approach continued in the twentieth century with many new policies, such as banning the addition of lead to gasoline, requiring that cars are fitted with seat belts and that these must be used, and prohibiting smoking in many public places. This strategy has been applied to the field of nutrition. Regulations were implemented decades ago in the area of food safety. Likewise, it became compulsory for particular micronutrients to be added to some foods. Examples include the addition of iodine to salt and of niacin to flour. In 1996, the USA and Canada implemented a policy that mandated the addition of folic acid to grain products. The goal was the prevention of spina bifida in infants. These policies have generally achieved a high degree of success. Moreover, the policies, in most cases, are highly cost-effective.

The USA and Canada implemented a policy that forced food manufacturers to remove trans-fatty acids from foods [21]. The motivation for this policy was the discovery that consumption of these fats increases the risk of CHD [22]. These unnatural fats are formed when oils are hydrogenated. Major foods sources include hard margarine, cakes, doughnuts, cookies, and deep-fried foods. Thanks to this policy, these fats have been largely eliminated from food sold in the USA and Canada. Other countries have followed a similar policy but with more modest targets.

Salt is another substance in food, where a strong case can be made for an action policy so as to reduce the intake by the population. However, in sharp contrast to the case of trans-fatty acids, governments have avoided implementing such a policy. The intake of salt in Western countries is typically around 8 to 12 g/day (3000–4500 mg of sodium per day). A mere 2% of the American population meets the recommended target for sodium intake (<2300 mg/day) [18]. This excessive intake plays an important role in the causation of hypertension [23, 24] and cardiovascular disease [24, 25]. Based on this evidence, there is a strong support for the view that a reduction of one-third in the salt content of food could help prevent many thousands of cases of cardiovascular disease [26, 27].

But how should this goal be achieved? The solution to this problem is well known: the quantity added to processed food must be greatly reduced as that is where roughly 75% to 80% of dietary salt comes from [28]. However, what is the most sensible way to achieve this goal? One approach is to rely on voluntary action. The UK followed this path. Starting in 2000/2001, the UK government embarked on a campaign to lower salt intake [29]. The major part of the campaign centered on voluntary action by industry. In the seven years following the implementation of the campaign (2001 to 2008), salt intake by adults in the UK fell by about 10% [30]. This accomplishment has been much praised. In reality, however, it achieved far less than the required one-third reduction in salt intake. There is little doubt that an action policy approach could have achieved the required reduction in salt intake much more effectively and much more speedily. Should the governments of the USA and other countries implement this policy? Considering the great potential for the prevention of thousands of needless deaths from CHD and strokes, to do otherwise would be heartless and a no-brainer!

There is a substantial literature on the relationship between food prices and sales. It is well established that a decrease in sales occurs when there is a rise in price (and vice versa). This is known as price elasticity. This rule of economics strongly supports the view that taxes and subsidies can be used as an action policy to achieve the desired effect on food consumption [31–33]. The weight of evidence indicates that a price change of 10–15% would achieve a significant effect on consumption [34].

There has been a widespread interest in a policy of adding an extra tax on sugar-sweetened beverages (SSB) in order to reduce sales. Such a policy could be extended to other sugar-rich foods. Scheelbeek et al. [35] recently analysed the likely impact on sales if the UK implemented a 20% price rise on sugar-rich snacks such as chocolate, biscuits (cookies), and cakes, but excluding SSB. The researchers concluded that this would result in a fall in mean BMI of 0.53 units. A notable conclusion was that the effect of the price rise on sales is more than double that of a similar price rise on SSB.

This action policy could be extended to other foods in order to help achieve healthier diets. A tax could be added to products containing refined grains while applying subsidies to fruit and vegetables and products containing whole grains.

A variation of this action policy is to provide people who are on a low income with vouchers that can be exchanged for healthy foods. Studies in both the UK [36] and USA [37] reported an increased in intake of fruit and vegetables when low-income women were given vouchers that could be exchanged for these foods. Some jurisdictions already have systems in place for giving vouchers to those in need. These are often referred to as “food stamps” in the USA. Implementing this action policy would require little more than a change of the rules governing how the food vouchers could be used.

Vast numbers of schools across North America have vending machines that sell unhealthy foods or beverages. This is a double problem: not only are schools complicit in facilitating the consumption of an unhealthy diet by their students but they are also delivering an implicit educational message “never mind about what you learned in the food guide, junk food is OK.” A recent review assessed the impact of various interventions such as ensuring that vending machines stock only healthy foods and that meals served in schools are of high nutritional quality [38]. The authors concluded that, on average, the impact of these interventions has been an increase in fruit consumption by 0.27 servings/day and a decrease in intake of SSB by 0.18 servings/day. Based on these various lines of evidence, there is a compelling case that schools should be compelled to restrict the sale of unhealthy food. Some jurisdictions actually take this issue seriously. For example, in 2011, Spain implemented a policy banning unhealthy food from schools [39].

## 5. Barriers to the Implementation of Action Policies

There are barriers that obstruct the path to the implementation of action policies. One is that governments are often extremely hesitant to implement radical new policies. Governments are often supportive of action policies that are barely noticed by the general population, such as making it compulsory for food manufacturers to change the composition of foods by the addition of particular micronutrients or the removal of trans-fatty acids. However, when the policy is much more obvious, such as the manipulation of food prices, then governments may need much prodding by advocates of public health.

A major barrier to the implementation of effective action policies is that there is likely to be stiff opposition from food corporations and their lobbyists. These corporations are emulating the game plan developed decades ago by the tobacco industry. That industry strongly opposed all efforts to increase taxes on cigarettes and policies that curtailed smoking in public places. Here is an illustrative example of how food corporations are likely to oppose action policies. As mentioned above, several jurisdictions in the USA and elsewhere have attempted to add a tax on sugar-sweetened beverages (SSB) in order to reduce sales. This has been repeatedly opposed by the corporations that manufacture SSB for the obvious reason that such taxes threaten their sales (and profits) [40]. It is therefore of utmost importance that those advocating for action policies are resolute.

## 6. Discussion

We now have the accumulated evidence from several decades of health promotion interventions that have employed a wide range of approaches to education, advice, and encouragement (EAE). The reduction in risk of CVD is often used as the yardstick of success as that disease is related to many components of the diet as well as to smoking and exercise. The degree of success of these interventions has been highly variable. Overall, they have the potential to cut risk of CVD by about 5 to 15%. The sorry state of the typical American diet, despite decades of admonitions by health experts regarding the importance of eating a healthy diet, exemplifies the inherent limitations of EAE.

Action policies by governments in the area of nutrition could, potentially, achieve much greater success. It is highly probable that the removal of trans-fatty acids from foods sold in the USA is already preventing thousands of cases of CHD. The same could be achieved if an action policy were implemented that mandated the removal of half the salt added to processed foods. Taxes on unhealthy foods, such as SSB, combined with subsidies on healthy foods, such as fruit and vegetables, could lead to a significant improvement in the diet of the general population.

We now have strong evidence that action policies are more effective than those based on EAE. Considerations of cost point to another advantage of action policies. Measured as cost per person reached interventions that rely on EAE and target people in the community, at the worksite, or in the physician's office are manpower intensive and are therefore expensive. Action policies, by contrast, can reach a large part of the population at a relatively modest cost. As a result, action policies are, in general, much more cost-effective [41].

There are clear signs that public health experts and governments are becoming increasingly aware of the strategy discussed here. This suggests that action policies, similar to those discussed here, are likely to play a greater role in health promotion, especially in the area of preventive nutrition.

It must be stressed that there are major limitations with much of the evidence referred to in this paper. The limitations of the evidence pertaining to EAE were discussed in the final paragraph of Section 3. This is also the case with the evidence that supports the value of action policies. In several cases, actual successes of action policies have been referred to. However, with other proposed action policies, the supporting evidence is based on indirect evidence and logical reasoning rather than on findings from actual case studies.

The adoption of a comprehensive action plan−“strategic nutrition”−designed to achieve a major advance in public health was previously proposed [42]. Strategic nutrition is, in essence, a combination of EAE and action policies. Component parts include the judicious use of taxes and subsidies on foods, restrictions on advertising, providing advice for the general population, and improved design of food guides and food labels. The arguments presented here are complementary to those made in that paper. The World Cancer Research Fund International made similar proposals with a plan known as nourishing [43].
